# Causes of Abortions in South American Camelids in Switzerland—Cases and Questionnaire

**DOI:** 10.3390/ani11071956

**Published:** 2021-06-30

**Authors:** Isabelle Rüfli, Corinne Gurtner, Walter U. Basso, Beatriz Vidondo, Gaby Hirsbrunner, Patrik Zanolari

**Affiliations:** 1 Clinic for Ruminants, Department of Clinical Veterinary Science, Vetsuisse-Faculty, University of Bern, 3012 Bern, Switzerland; isabelle.ruefli@vetsuisse.unibe.ch (I.R.); gaby.hirsbrunner@vetsuisse.unibe.ch (G.H.); 2 Institute of Animal Pathology, Department of Infectious Diseases and Pathobiology, Vetsuisse-Faculty, University of Bern, 3012 Bern, Switzerland; corinne.gurtner@vetsuisse.unibe.ch; 3 Institute of Parasitology, Department of Infectious Diseases and Pathobiology, Vetsuisse-Faculty, University of Bern, 3012 Bern, Switzerland; walter.basso@vetsuisse.unibe.ch; 4 Veterinary Public Health Institute, Vetsuisse-Faculty, University of Bern, 3012 Bern, Switzerland; beatriz.vidondo@vetsuisse.unibe.ch

**Keywords:** South American camelid, llama, alpaca, abortion, stillbirth, *Coxiella burnetii*, Switzerland

## Abstract

**Simple Summary:**

South American camelids (SAC) are increasingly popular in Switzerland for keeping as pets. For many farmers, SACs are a supporting leg when offered for trekking. Products made from alpaca fibers are in demand, and even the market for meat is increasing as they are extensively hold. Low reproductive rates and abortions/stillbirths are one of the main complaints among alpaca and llama breeders. The purpose of this study was to investigate the reasons for pregnancy loss and perinatal death in SAC herds in Switzerland. Aborted and stillborn fetuses were collected, necropsied and analyzed for infectious causes. This was complemented with a nationwide survey among breeders. During a 1.5-year period, eight cases of aborted or stillborn alpaca and llama have been analyzed. For the first time, the bacterium *Coxiella burnetii,* a known cause of abortions and stillbirths in small ruminants and cattle, could be detected in aborted alpacas and llamas. As this pathogen has the possibility to infect humans (=zoonotic potential), it is important to gain more knowledge about its significance among SAC. This study has set the ground stone for further investigations about abortifacients in Swiss SAC herds.

**Abstract:**

Over the last decade, South American camelids (SAC) have gained increasing popularity in Switzerland. They are used for several purposes such as fiber and meat production, as companion or guard animals and for trekking activities. The purpose of this study was to investigate the frequency and reasons for pregnancy loss and perinatal death in SAC herds. Within the scope of this study, early embryonic losses could not be identified, as pregnancy examinations by ultrasonography are not performed routinely. Aborted and stillborn fetuses were collected, necropsied and analyzed for infectious abortifacients. A nationwide survey among breeders was carried out. During a 1.5-year period, only eight cases of aborted or stillborn alpaca and llama (out of a population of 6550 animals) were reported by the breeders, and their causes were subsequently analyzed. In half of the cases, *Coxiella burnetii* was identified in the fetoplacental material. Abortions and stillbirths were reported to be rare in Swiss herds. As a conclusion, recording of embryonic losses through ultrasound training of veterinarians should be impaired and breeders motivated to have abortions and perinatal mortality examined. Special focus should be laid on *C. burnetii* due to its zoonotic risk.

## 1. Introduction

Raising healthy offspring plays a vital role in South American camelid husbandry. Thus, fetal and perinatal loss are amongst the most important factors contributing to lowered reproductive efficiency and one of the major complaints amongst camelid North American breeders [1,2]. Birth rates of 50% and 45.9%, respectively, in alpaca (*Vicugna pacos*) and llamas (*Lama glama*) have been described, pinpointing early pregnancy loss (50–57.8%) as one of the main factors contributing to the low fertility of SAC [1]. Most embryos (50%) seem to be lost in the first month [3]. Camelid reproductive physiology exhibits some differences compared to other ruminants. Ovulation in camelids only takes place upon induction [4]. Gestational length in SAC ranges from 342 to 350 days [5]. Normal cria weight at birth ranges from 6.7 to 9.1 kg in alpacas and 10.2 to 13.6 kg in llamas [6].

Previous studies in New Zealand detected an abortion rate of 5–17% [7,8]. Other studies have shown that finding the causative agent for the abortion can be difficult, leading to a rather big proportion of cases where the reason stays unknown [9,10]. Abortion is defined as the expulsion of a fetus after organogenesis is completed and before fetal independent viability [11]. Stillbirth describes the death of the mature fetus before or under parturition [11]. Known reasons for abortion and stillbirth can be categorized into two main categories: infectious and non-infectious causes. Infectious abortifacients can be of viral, bacterial or parasitic nature. Bovine viral diarrhea virus (BVD) is one of the most common viral abortifacients found in SAC [10,12]. Bacteria are another important group of infectious organisms provoking abortions and stillbirths. So far, *Brucella*, *Campylobacter fetus* subsp. *fetus* spp., *Chlamydophila* spp., *Leptospira* spp., *Listeria monocytogenes*, *Salmonella* spp. and *Trueperella pyogenes* have been isolated from aborted SAC fetuses [1,10,13,14,15,16]. Additionally, parasites may cause abortions and stillbirths in SAC; namely, *Neospora caninum* and *Toxoplasma gondii* are known parasitic abortifacients [1,17].

Early embryonic death is the most common form of reproductive loss in SAC, estimated to affect 10–15% of all pregnancies in the first 60 days of pregnancy [18]. In extreme conditions, up to 60–80% in the first 90 days of gestation might occur [18].

Twin pregnancies, stress, umbilical torsion and placental insufficiency account for the non-infectious causes of abortion and perinatal mortality [4,19]. The definition of perinatal mortality varies among studies and is in some cases used as a synonym of stillbirth [20]. Usually, perinatal mortality refers to death at full term up to 48 h after parturition [21]. Perinatal mortality accounted for 22.4% alpaca and 8.6% llama deaths in the UK SAC population [22]. The most common causes of perinatal mortality were a lack of colostrum and hypothermia [6,22]. The age of the dam also has an impact on perinatal survival of the cria. Younger dams were found to have higher perinatal loss (11.3%) of their crias, than more mature dams (5.9%) [6]. Bravo et al., 2009, hypothesized that younger dams give birth to smaller and lighter crias [6]. The smaller size of the crias means increased difficulty in reaching the udder, which leads to higher risk of perinatal death due to starvation and lack of colostrum. South American camelids are popular in Switzerland [23]. The number of alpacas increased by 4.3% in the year 2020, whereas the llama population decreased by 1%, according to the Swiss Federal Office for Statistics [24]. In 2019, the SAC population counted 2882 llamas and 3668 alpacas in Switzerland [25]. The animals are used in a variety of purposes such as fiber and meat production, hobby, breeding or as guard animals [23,26]. At the level of the Swiss legislation, SAC have production animal status [27]. Camelid husbandry in Switzerland differs to that in South America, and, hitherto, the frequency and reasons for abortions and perinatal mortality in Switzerland might also differ.

The objective of this study was to investigate the causes and to gain knowledge about the impact of abortion, stillbirths and perinatal mortality in Swiss SACs. In a first part of the study, carcasses of aborted, stillborn and perinatally diseased crias were collected over a 1.5-year period (2018–2020). Subsequently, necropsies were performed, and samples taken and analyzed for known abortifacients. Jugular vein blood was taken from the dams for determination of antibodies and trace element status. Currently, there are no data on trace element values in SAC in Switzerland. In the second part of the study, breeders were sent a questionnaire to gain information about Swiss SAC husbandry and how often breeders have to deal with pregnancy loss and perinatal mortality.

## 2. Materials and Methods

This investigative model involved: (1) recruitment of SAC herds with abortions, (2) collection of samples (fetus/cria, placenta, serum of the dam) from these herds and distribution of questionnaires to the owners, (3) necropsy and laboratory examinations of the samples collected and (4) distribution of a nationwide questionnaire about reproductive losses to SAC owners.

### 2.1. Field Samples from South American Camelids and Farm Data

Aborted crias were collected throughout Switzerland during a 1.5-year (July 2018–February 2020) sampling period. Alpaca and llama breeders were informed about the project by means of a short article in a magazine for breeders distributed by the Swiss Counseling and Health Service for Small Ruminants. Furthermore, a short description of the project was presented in a nationwide continuing education program for food animal practitioners in Bern in June 2018. Additionally, veterinarians and breeders were informed about the project at a congress about SAC reproduction in April 2019. The fetoplacental unit was retrieved within a day of the notification by the owners and then kept in cold storage (4 °C) until immediate necropsy. Simultaneously, blood samples [S-Monovette^®^ (no anticoagulant), 10 mL Luer tubes, Sarstedt, Nümbrecht, Germany] were taken from the jugular vein of the dam. The blood samples were centrifuged within 4 h of collection at 3500 revolutions per minute for 10 min (Hettich^®^ EBA 20centrifuge, Hettich, Switzerland) and the sera stored in Eppendorf tubes. One sample was immediately sent to the Institute of Veterinary Bacteriology (Vetsuisse-Faculty, University of Bern, Switzerland) for serologic analysis of brucellosis, a second tube was sent to the Institute of Parasitology (Vetsuisse-Faculty, University of Bern, Switzerland) for serology of neosporosis and toxoplasmosis, a third sample was sent to the Institute of Virology and Immunology (Vetsuisse-Faculty, University of Bern, Switzerland) for Bovine Herpes Virus 1 (BHV-1) serology, and a forth tube was stored at −20 °C for trace element analysis. The owners were asked to fill in a questionnaire with 50 questions. The questionnaire consisted of a general part, asking questions about the farm structure and management, and a specific part, inquiring about the abortion, health status of dam and sire and recent management changes. Between July 2018 and February 2020, a total of eight aborted alpacas and lamas and their corresponding placenta and serum of the dam were retrieved. All animal experimentation was performed with permission and in accordance with Swiss law. The following approval number was allocated by the animal experimentation commission (elected by the cantonal executive counsel) BE 30/18. All participating breeders gave written consent for all examinations.

### 2.2. Necropsy

The fetuses were necropsied at the Institute of Animal Pathology (Vetsuisse-Faculty, University of Bern, Switzerland) at the latest within 48 h of parturition. First the weight of fetus and placenta were determined, as well as the crown–rump length (CRL, distance between the external occipital protuberance and the end of the sacral bone using a measuring tape) of the fetus. Fetal gender was assessed, and the body was searched for malformations. The carcass was dissected according to standard operation protocols [28,29], and laryngeal muscles, lung, heart and liver tissue were collected and fixed in 10% formalin for histologic examination. These tissue samples were paraffin-embedded and processed to hematoxylin and eosin-stained tissue sections. For microbiological analysis, an ear notch sample, tissue samples of the myocardium, liver, lung, as well as a brain hemisphere, the ligated compartment three and body fluid of the fetus were collected. The placenta was controlled for completeness and macroscopic lesions. Tissue samples were retrieved for microbiologic examination, and a piece was fixed in 10% formalin for histologic analysis, taking care not to collect a part from the physiologically avillous area. The histopathological tissue slices were evaluated by board-certified pathologists for the presence of inflammatory and degenerative lesions.

### 2.3. Laboratory Testing

#### 2.3.1. Parasitological Analysis

Total DNA was extracted from fetal brain, myocardium and placenta samples at the Institute of Parasitology (Vetsuisse-Faculty, University of Bern, Switzerland) as described [30], and real-time PCRs to detect *N. caninum* [31] and *T. gondii* DNA [32] were performed. In addition, serum samples of the dam and fetal body fluids were analyzed by commercial enzyme-linked immunosorbent assays (ELISA) to detect antibodies against *N. caninum* and *T. gondii* [33].

#### 2.3.2. Virological Analysis

Bovine viral diarrhea virus was detected by ELISA, performed on fetal ear notch samples sent to the IDEXX Diavet laboratory (Bäch, Switzerland). The commercial IDEXX BVDV Ag/Serum Plus kit (IDEXX Switzerland, Liebefeld-Bern, Switzerland) was used [34]. In order to detect antibodies against BHV-1, serum samples of the dam were analyzed with antibody ELISA IDEXX IBR gB Ab Test (IDEXX Switzerland, Liebefeld-Bern, Switzerland) according to international standards [35]. Placenta, fetal lung and liver tissues were analyzed at the Institute of Virology and Immunology (Vetsuisse-Faculty, University of Bern, Switzerland) by direct immunofluorescent detection of equine herpes virus 1 (EHV-1) antigens according to OIE standards [35].

#### 2.3.3. Bacteriological Analysis

Serum samples of the dam as well as placenta, fetal lung, liver and the ligated compartment three were all analyzed at the Institute of Veterinary Bacteriology (Vetsuisse-Faculty, University of Bern, Switzerland).

##### Broad-Spectrum Culture

Broad-spectrum bacterial and fungal culture was performed for all samples of placenta, fetal lung, liver and compartment three content.

##### Staining and Microscopy

All tissue samples were evaluated with the Stamp’s modification of the Ziehl–Neelsen staining [36].

##### Molecular Detection of Bacteria

In order to detect *Coxiella burnetii* and *Chlamydia abortus*, total DNA was extracted as described [30]. Subsequently a qPCR was performed [37,38].

##### Serology

*Brucella abortus* and *Brucella melitensis* antibodies were detected using the IDEXX Brucellosis Serum X2 test kit (IDEXX Switzerland, Liebefeld-Bern, Switzerland).

#### 2.3.4. Trace Elements Analysis

Frozen serum samples (stored at −20 °C) of the dam were used to determine their trace element levels at the time of abortion or stillbirth. The samples were collected, frozen and then sent in bulk to be analyzed at an external laboratory (Labor Zentral, Geuensee, Switzerland). Zinc and copper values were detected by means of photometry. Inductively coupled plasma mass spectrometry was used to identify serum selenium and iodine concentration. In order to detect manganese, an atom absorption spectroscopy was performed.

### 2.4. Questionnaire

In order to assess putative risk factors and gain a general overview of how often owners experienced abortions in their herd, a standardized web-based questionnaire was sent to all the SAC members of the Swiss Counseling and Health Service for Small Ruminants (*n* = 377) by email. The questionnaire contained 12 questions including general questions about the farm (i.e., SAC species, herd size, other animals on the farm), management (who takes care of the animals, purchase) and occurrence of abortions and stillbirths (Supplementary Materials Questionnaire S1 and Questionnaire S2). Answering of the questionnaire was voluntary, and all collected data were anonymous.

### 2.5. Statistical Analysis

The continuous variables (gestation length, body weight of fetuses, placental weight, CRL, maternal blood trace element concentration, age of the dam) were described using median and quartiles. The categorical variables (breed, purchase, other animals present, defined necropsy findings, presence of pathogen infection) were described using frequencies and proportions. Due to the limited number of cases, the statistical analysis was limited to descriptive statistic only. Data were analyzed using the statistical software NCSS 11 (2016) (NCSS, LLC., Kaysville, UT, USA).

## 3. Results

### 3.1. Field Samples of Abortion Cases

#### 3.1.1. Farm Characteristics

The eight cases of aborted/stillborn crias originated from seven Swiss farms. One farm submitted two abortions of the same dam in two consecutive gestations. The seven participating farms were all located in the German-speaking part of Switzerland in the cantons of Bern (four cases on three farms), Lucerne (two cases), Uri (one case) and Zurich (one case). All herds were taken care of by members of the family. The mean herd size was 25.5 animals with a minimum of five animals and a maximum of 65. All farms kept their animals in a combination of pasture and barn. More than half of the participating farms (5/8) kept other animals such as cattle (*n* = 3), cats (*n* = 3), dogs (*n* = 2) or chicken (*n* = 2) on their premises.

#### 3.1.2. Data about the Dam, Cria and the Abortion/Stillbirth

Of the eight cases included in this study, six of the dams were alpacas, while two were llamas. The mean age at the time of abortion or stillbirth was eight years, with the youngest dam being four years and the oldest 13 years old. The majority of the dams (5/8) were healthy at the time of abortion. One dam was diagnosed with *Mycoplasma haemolamae* and endoparasitosis prior to giving birth. A second one showed a swelling at the level of the mandible ante partum, whereas a third dam was ill of unknown cause at the time of parturition. Three of eight dams had a history of pregnancy loss in a previous gestation.

The fetuses were expelled at a mean of 306 days of gestation. In two cases, the gestational age was unknown, because the owner had not noted the exact day of mating. The majority of fetuses were female (*n* = 5). Most of the offspring were aborted without any assistance. In two cases, the owner helped the dam expel the fetus. Three crias were stillborn. Only one parturition took place during the night, while the majority of dams (7/8) gave birth during daylight hours.

#### 3.1.3. Necropsy and Histopathological Findings

The mean placental weight, fetal weight and CRL were 1.3 kg, 5.6 kg and 59.5 cm, respectively. More details concerning weight, CRL and placental weight are shown in Table 1. None of the aborted fetuses showed signs of malformation. The following observations were made during necropsies: fetal pulmonary atelectasis (8/8), bronchopneumonia (1/8), macroscopically visible (and/or histologically) visible meconium (3/8), placental mineralizations (4/8), placental edema (1/8), placental necrosis (1/8) and placentitis (3/8). In one case of placentitis, bacterial colonies were seen histologically on the surface of the chorion, as well as infiltrating into the chorion.

#### 3.1.4. Serological and Microbiological Findings

Pathogen detection and serology results are shown in Table 2.

Antigens/DNA of BVDV, EHV, *Neospora caninum*, *Toxoplasma gondii*, *Brucella abortus*, *Brucella melitensis*, *Campylobacter foetus* subsp. *Foetus*, *Campylobacter foetus*, subsp. *Venerealis*, *Chlamydia abortus*, *Salmonella* sp. and pathogenic *Leptospira* spp. have not been detected. Antibodies against BHV-1, *Neospora caninum*, *Brucella abortus* and *Brucella melitensis* have not been detected. In all dams, antibodies against *Toxoplasma gondii* were found.

Direct evidence of infection (Ag-ELISA, culture, PCR in combination with histopathology results) was found in 5/8 animals. There was one co-infection found in a dam with *C. burnetii* and *Strep. pluranimalium*. The dam had a history of already having aborted in a previous gestation. Another dam aborted twice in a row, and both fetuses were submitted to this study. In both cases, *C. burnetii* could be detected by qPCR. Despite detailed case histories and extensive necropsy and laboratory examinations, the ultimate cause of death (UCOD) could not be assigned in 3/8 cases.

Indirect evidence of exposure to an infectious agent (Ab-ELISA) was found in all dams. All of the tested females had developed antibodies against *T. gondii*. Although *T. gondii* DNA could not be detected in any of the fetuses or corresponding placentae by PCR, antibodies against *T. gondii* were detected in one aborted fetus (Table 1 and Table 2: No. 8) and one stillborn cria (Table 1 and Table 2: No. 7) indicating infection.

#### 3.1.5. Trace Mineral Analysis

In two animals (dams 1 and 2), the serum selenium level was low, in four animals (dams 2, 3, 4 and 6) the zinc level was low, respectively. Trace mineral status of all the dams are shown in Table 3.

### 3.2. Questionnaire

The questionnaire with 12 questions about herd management and experiences with abortions and stillbirth was sent to 377 members of the Swiss Counseling and Health Service for Small Ruminants. The questionnaire was returned by 43 members (11.40%), fully completed by 32 members (8.48%), while 11 questionnaires were only partially completed (2.91%), four questionnaires being submitted empty (1.06%).

The majority of the participating herds (93%) are taken care of by family members. About half of the farms (53.1%) stated to have 1–5 dams, whereas 25% have 6–10 dams, 9.4% 10–20 dams and 12.5% more than 20 dams in their herd. Keeping other animals beside SAC seems to be quite common, with 61% of the participants answering to keep another species. Companion animals being the most popular. Cats were mentioned as pets in 48.7% of households and dogs in 38.5% of homes. Cattle were kept alongside 23% of SAC herds, whereas horses were only present in 12.8% of participating farms. Other animals mentioned to live with alpacas and/or llamas were small ruminants (*n* = 8), poultry (*n* = 8), donkeys (*n* = 2) and rabbits (*n* = 2). Most of the purchased SAC originate from Swiss farms (71%). None of the farms purchased animals from North or South America. One in 39 herds each made acquisitions in Europe or Australia and New Zealand. In 11 of 26 herds, the new arrivals were isolated prior to introduction into the existing herd. The majority of breeders (19/27) stated that no abortion occurred in the previous two years. In six (22%) herds, one abortion had happened in the previous two years, and in two herds (7%), even two abortions occurred. Only 36% answered the question if they experienced a stillbirth in the previous two years. One stillbirth had occurred in nine (64%) herds, two stillbirths in two (14%) herds, whereas three (21%) breeders answered that they had never lost a cria due to stillbirth in the previous two years. In summary, this results in a total of 19 (49%) reproductive losses during the previous two years, either in the form of abortion or stillbirth, amongst the participating owners. Only 3.6% (1/28) breeders separate the dams for parturition. Parturitions were observed by the majority of breeders (21/27).

No evidence of significant association was found with herd size, purchase, other animals present and abortion or stillbirth, respectively.

## 4. Discussion

In the 1.5-year field sample collection period, only eight cases of abortion or stillbirth could be collected and necropsied from a total population of 6550 animals. Considering the reported 10% of abortions in SAC in a recent survey in Switzerland, Austria and Germany of [40], one could expect 655 cases per breeding season. Other authors described difficulties in gaining access to cases due to remote farms and great distances to travel [41], which is less of a problem in Switzerland. The number of abortions reported in the seven Swiss farms was similar to the number of cases included in our study. With the data at hand, it is not clear whether abortions really are a minor concern in Swiss SAC herds or if the herds with more losses did not participate in our study. By legislation, SAC abortions do not have to be examined. This is probably why we have such low numbers. Despite strong advertising for this pilot study at veterinary conferences, in journals, newsletters and the information that we come to collect the abortions and that everything is free of charge, we were not able to acquire more abortions for pathological examination over the defined period. In Swiss SAC herds, ultrasonographic pregnancy diagnoses are not routinely undertaken. Therefore, no statement on early embryonic losses is possible. Statistics on abortions in Switzerland only concern those detected visually by owners. Further investigations are necessary to elucidate the prevalence and cause of abortions in Switzerland.

All dams that reported abortions or stillbirths in the current study were seropositive for *T. gondii*. This is in accordance with a previous seroprevalence study in Switzerland, showing 83.2% of Swiss SAC to be seropositive for the parasite [33]. In the questionnaire of our study, cats were reported as the most popular pet to be kept alongside alpacas and llamas. This fact leads to a higher probability to have contact with *T. gondii* and becoming seropositive. In the study of Basso et al., 2020, “absence of cats in the last two years” was found to be a protective factor [33]. From none of the aborted or stillborn fetuses of the seropositive dams, *T. gondii* DNA could be detected by qPCR. However, one aborted fetus and one stillborn cria showed antibodies against *T. gondii* in their body fluid. As maternal antibodies do not cross the placental barrier in SAC, this indicates that those fetuses got infected with *T. gondii* during pregnancy. If the infection was also the cause of the pregnancy, loss remains unclear. We could assume that the dam became primo-infected during pregnancy and transmitted the parasite vertically to the fetus. Stillbirth caused by *T. gondii* has so far been described once, in which case *T. gondii* tissue cysts could be found in both kidneys, along with mild non-suppurative lesions in the placenta, liver and heart [42].

In five of eight of the aborted or stillborn fetoplacental units, *C. burnetii* could be isolated from fetal organs, the placenta or both. Only in three cases, corresponding histological lesions were found as placentitis and another case revealed edema of the lamina propria of the placenta. Two of the corresponding dams had a history of a previous pregnancy loss. To our knowledge, it is the first report of an abortion caused by *C. burnetii* in a SAC. *C. burnetii* is a well-known cause of abortion in small ruminants [43,44]. In a Swiss prevalence study among small ruminants, *C. burnetii* could be isolated in 44.4% of aborted sheep and 44.2% of aborted goats [45]. The numbers are in line with the publication of [30] with submitted abortion material during the years 2012–2016 in sheep with 46.3%, respectively, in goats with 52.8%. In a Swiss study on PM in cattle, *C. burnetii* was detected in 32% of calves by qPCR, but only 15% of placentae and 19% of calves had histological signs of inflammation (placentitis and bronchopneumonia), respectively [20]. The authors suggested that acute infections with *C. burnetii* might occur without histological lesions [20]. Regarding the zoonotic potential of *C. burnetii* and the high numbers of bacteria shed after an abortion [45,46], it is essential to investigate abortion cases in SAC in order to implement protective measures for the owners and the remaining animals in the herd.

One of the aborted fetuses showed histological signs of a severe bronchopneumonia. Microbiological analysis isolated large amounts of *E. coli* in the fetal liver, lung and compartment three, as well as in the placenta, additionally to *C. burnetii* detected in the placenta by PCR. These findings might point to an opportunistic infection or a co-infection leading to the stillbirth of the animal. Normally, as also in this case, the mother shows no clinical signs. Opportunistic infections with *E. coli* leading to stillbirth are also described in cattle [47] and humans [48].

In two cases where no infectious agent could be detected in the fetus, the corresponding dams showed signs of systemic illness, one dam aborted after transport. In these cases, we regard the systemic illness or stress to be the reason for the pregnancy loss in these dams. Additionally, others have made the same observation that illness of the dam led to abortions with no microbiological or pathological findings [49,50].

Regarding the existing knowledge about abortions and perinatal mortality in SAC, the available literature is scarce. The majority of available publications originates in North America, and the question remains what the situation in Europe is like. There are still large gaps of knowledge in terms of which infectious agents are of significance in SAC in Switzerland and Europe. BVD proofed to be the most frequently studied infectious agent. Additionally, results of the studies show that infections with BVD can lead to abortions and stillborn fetuses, but the highest risk lies in the development of persistent infected (PI) animals, which can spread the virus more effectively [12,51,52,53]. Especially in SAC, the risk of spreading BVD with PI crias is serious, due to the often seen habit of taking dams and their suckling cria to other farms to be bred [53,54]. When Switzerland started its BVD eradication program, a study about the impact of SAC in spreading the virus was performed [55]. A prevalence rate of 5.75% for BVD in the year 2008 was found. Which led to the conclusion that SAC play no major role in the spread of BVD in Switzerland, and thus, SAC were not included in the BVD eradication program of Swiss cattle [55]. Apart from the studies about BVD, there are only isolated case reports about other infectious abortion pathogens. Infectious causes described in case reports include: EAV [56], *T. gondii* [42], *N. caninum* [17,41], *Sarcocystis* [57], *L. monocytogenes* [14], *C. fetus fetus* [16], *Strep. sanguinis* [58] and *Coccidioides posodasii* [59]. Regarding the zoonotic risk of some of the abortifacients and the popularity of SAC and the close contact to humans, it is of great importance to keep track of occurring abortions in SAC herds. Furthermore, it is vital to sensitize SAC owners to notify their veterinarian about abortions and send the appropriate material to laboratories for further investigation of the etiology of the abortion and taking appropriate safety measures.

As prepartum maternal dietary trace element imbalances (selenium, zinc, copper, iodine) are associated with PM in calves [60], trace minerals were analyzed in dams, especially as no data in SACs are available in Switzerland. In two dams, selenium values were below indicated reference value [14], but no corresponding histologic pathologies could be seen. The selenium findings may be explained by the low selenium status of Swiss soils [61]. Low zinc values were described in mummification and abortion in ewes [62], but the low values found here may be incidental.

Concerning perinatal mortality, the review revealed a wide variety of definition, due to which not many publications met our inclusion criteria. There is a correlation between the age of the dam and the chance of survival of the cria [6,63]. This suggests that it is useful to monitor young dams around parturition to ensure that the offspring receive sufficient colostrum and, hence, have a higher chance of survival.

## 5. Conclusions

With the data at hand, it is not clear whether abortions really are a minor concern in Swiss SAC herds or if the herds with more losses did not participate in our study. Gaining sufficient data by voluntary participation proved to be a major challenge. Further information campaigns, incentives and prevalence studies are necessary to obtain a more precise picture of reproductive losses in Swiss herds. Recording of embryonic losses through ultrasound training of veterinarians should be impaired and breeders motivated to have abortions and perinatal mortality examined. For the first time *C. burnetii* could be identified as an abortifacient in SAC, and our results indicate that it should be considered as a differential in cases of abortion.

## Figures and Tables

**Table 1:** Necropsy findings in 8 cases of abortion or stillbirth in 7 Swiss South American camelid herds.

Fetus	CRL	Fetal Weight	Placental Weight	Completeness of Placenta
	[cm]	[kg]	[kg]	
	Reference: [19]	Reference: [6]	Reference: [39]	
Alpaca	47–66	6.7–9.1	0.61–0.99	
Llama	57.1–78.7	10.2–13.6		
Fetus 1	52	3.2	0.42	Complete
Fetus 2	32	0.49	0.25	95%
Fetus 3	55	2.94	1.96	Complete
Fetus 4	46	2.1	0.56	Complete
Fetus 5 *	84	10.6	2.6	Complete
Fetus 6	66	9.6	2.84	Complete
Fetus 7 *	85	11.4	0.96	Complete
Fetus 8	56	3	0.8	Complete

* llama fetus; CRL = crown–rump length.

**Table 2:** Pathogen antigen and antibody detection and histologic findings in 8 cases of abortion or stillbirth in 7 Swiss South American camelid herds.

Fetus Alpaca Llama	Antigen Detection	Histologic Findings	Final Diagnosis of Abortion
Fetus 1	*negative*	complete atelectasis; no signs of placentitis	DNR (dam ill)
Fetus 2	*Mucor ssp* (P)	complete atelectasis; diffuse placentitis	*Mucormycosis*
Fetus 3	*C. burnetii* (P,A)	complete atelectasis; diffuse placentitis	*Coxiellosis*
Fetus 4	*C. burnetii/Strep. pluranimalium* (P,A)	partial atelectasis, meconium aspiration; disseminated placental necrosis	Co-infection (*C. burnetii/Strep. pluranimalium)*
Fetus 5 *	*C. burnetii* (A)	complete atelectasis and lung edema; placental edema	DNR (abortion after transport)
Fetus 6	*negative*	complete atelectasis	DNR (dam ill)
Fetus 7 *	*C. burnetii* (P) *E. coli* (Li,L,C3)	complete atelectasis, bronchopneumonia; no signs of placentitis	Co-infection (*C. burnetii/E. coli)*? [20])
Fetus 8	*C. burnetii* (P)	complete atelectasis; placentitis	*Coxiellosis*

* llama fetus; P = placenta; A = abomasum; Li = liver; L = lung; DNR = diagnosis not reached.

**Table 3:** Trace mineral status of 8 dams in 7 Swiss South American camelid herds.

Dam	Selenium	Iodine	Manganese	Zinc	Copper
	[umol/L]	[umol/L]	[nmol/L]	[umol/L]	[umol/L]
	Reference: [13]	Reference:	Reference: [13]	Reference: [13]	Reference: [13]
Alpaca	1.61–3.43	Not Available	12.74–198.4	2.9–31.35	5.5–12.43
Llama	0.94–3.30		12.74–87.37	3.21–17.28	6.29–12.43
Dam 1	**0.68**	0.19	18.93	3.06	9.94
Dam 2	**0.21**	0.22	37.31	**2.45**	9.7
Dam 3	1.92	1.3	23.48	**<1.53**	8.71
Dam 4	1.82	0.99	24.02	**2.75**	7.65
Dam 5 *	1.89	0.45	33.49	3.06	8.79
Dam 6	2.2	0.67	54.96	4.74	14.54
Dam 7 *	0.96	0.58	21.48	3.98	11.63
Dam 8	2.26	1.11	40.4	**2.75**	10.57

* llama dam; low reference values marked in bold.

## Data Availability

The datasets generated and/or analyzed during the current study are not publicly available due to the individual privacy of SAC owners but are available from the corresponding author on reasonable request.

## Supplementary Materials

The following are available online at https://www.mdpi.com/article/10.3390/ani11071956/s1. Questionnaire S1: Farm and Management Data, Questionnaire S2: Additional questions.
