# Supplementary material for: Causes of Abortions in South American Camelids in Switzerland—Cases and Questionnaire
Source: Animals (Basel). 2021 Jun 30;11(7):1956. doi: 10.3390/ani11071956 (PMC8300385; doi:10.3390/ani11071956)
Supplement: Supplementary file 1 [file animals-11-01956-s001.zip › animals-1248084-supplementary/Ethical authorization_Rüfli et al.pdf]

Geschichte der Änderungen

Abschnitt Nr. 54.1 in Method chapter 1_de was questioned_de on_de 09.04.2018 09:34
Abschnitt Nr. 54.1 in Method chapter 1_de was answered_de on_de 11.04.2018 17:41
Remarks added for section_de 51.1 in Method chapter 1_de on_de 11.04.2018 17:45
Remarks added for section_de 54.2 in Method chapter 1_de on_de 11.04.2018 17:48
Remarks added for section_de 55.0 in Method chapter 2_de on_de 11.04.2018 17:49

Datum Stempel

Ablauf: APPROVED Datum: 11.06.2018 2:31 AM

Gesuch für Tierversuche

Art.18 Tierschutzgesetz(TSchG), Art.141 Tierschutzverordnung(TSchV),Art.30 Tierversuchsverordnung(TVV)

1 Adresse der Bereichsleiterin/des Bereichsleiters (Institut, Firma)

Patrik Zanolari
patrik.zanolari@vetsuisse.unibe.ch
031 631 23 44
Wiederkäuerklinik
Departement für klinische Veterinärmedizin
Bremgartenstrasse 109a, REF-615-21
CH - 3012
Bern

2 Adresse der kantonalen Behörde

BE Veterinärdienst des Kantons BE, Sekretariat Tierversuche
Herrengasse 1
CH - 3011 Bern

3 TITEL DES PROJEKTS

Ursachen für Aborte und perinatale Sterblichkeit (Totgeburten) bei Neuweltkameliden in der Schweiz

Subtitle of experiment/project_ge

`Untersuchung und Beschreibung der Ursachen in Betrieben in der Schweiz`

31 Fachgebiet bzw. Anwendungsbereich:

32 Gesuchstyp

[] [N] neues Gesuch

33 Tierart

.

TIERART **Various mammals**	Gesamtzahl pro Gesuch 300	Herkunft/n (a-c) *Andere Herkunft, welche: Hoftiere*
Namen und Adressen der Lieferanten:<br>100 verschiedene Praxisbetriebe<br>Kanton<br>Kanton<br>alle<br>Switzerland		

34.1 Tierhaltungsort

_

TIERART	Adresse Tierhaltungsort:
**Various mammals**	100 verschiedene Praxisbetriebe<br>-<br>-<br>-<br>Switzerland

34.2 Adresse Durchführungsort:

Diverse Betriebe in der Schweiz.

34.3 Interkantonaler Versuch: Yes

Wenn ja, welche(r) anderer/n Kanton(e):

NE

LU

OW

SG

SH

SO

SZ

TG

TI

UR

VD

VS

ZG

ZH

AG

AI

AR

BL

BS

FR

GE

GL

GR

JU

NW

34.4 Verwendung von gentechnisch veränderten Tieren:
No

35 Maximaler prospektiver Schweregrad: (Details: siehe unter Ziff. 56.4)
0

36 Dauer des Projekts
Jahre: 3 / Monate: 0 / Tage: 0

Datum des geplanten Beginns
01.05.2018

37 Liste der Personen, die Massnahmen und Eingriffe im Rahmen des Versuches durchführen oder leiten:

Name der Person	Aus-/Weiterbildungsstand	Rolle im Versuch	Eingriff
Gaby Hirsbrunner	(Orange)	Deputy Study Director	Vertretung von Patrik Zanolari bei Krankheit, Abwesenheit
Patrik Zanolari	(Grün)	Study director	Organisation, Koordination, Unterstützung der Doktorandin
der Wiederkäuerklinik Ausgebildete Tierärzte und Tierärztinnen	(Grün)	Involved person	Betriebsbesuche, Probenentnahmen

38 Unterzeichnende/r Bereichsleiter/in — Last Submitted_ge
Patrik Zanolari — 11.04.2018

39 Unterzeichnende/r Versuchsleiter/in — Last Submitted_ge
Patrik Zanolari — 11.04.2018

Stellvertretende/r Versuchsleiter/in
Gaby Hirsbrunner

4 ANGABEN ZUR ZIELSETZUNG DES PROJEKTES (für die Statistik Art. 147 TSchV); Ziff. 41-43 ausschliesslich je einmal ankreuzen und ggf. bei den Detailfragen eine weitere Marke sowie Ergänzungen anbringen.

41 Das Vorhaben/ der Versuch steht in Zusammenhang mit
[] Krankheitsdiagnostik

42 Das Vorhaben steht in Zusammenhang mit

[] Krankheiten beim Tier. Welche:Abortursachen, perinatale Sterblichkeit (Totgeburten) bei Neuweltkameliden (Management, infektiös, genetisch)

43 Das Vorhaben steht in Zusammenhang mit gesetzlich vorgesehenen Verfahren (Registrierungs- und Zulassungsvorschriften):

[] Das Vorhaben/ der Versuch hat keinen Zusammenhang mit gesetzlich vorgesehenen Verfahren.

44.1 Allgemeine Beschreibung des Versuchsziels, Stand der Forschung, Darlegung, was noch nicht hinreichend bekannt ist (beispielsweise Zusammenfassung des NF -Gesuchs, maximal eine Seite):

Aborte können während der ganzen Trächtigkeitsdauer von 335 bis 360 Tagen auftreten. Unter perinataler Sterblichkeit bei Neuweltkameliden wird der Tod vor, während und bis 48 Stunden nach der Geburt verstanden. Gründe für Aborte und perinatale Sterblichkeit können im Management liegen (Fütterung, Versorgung mit Mengen- und Spurenelementen, Hygienevorkehrungen, Kontrolle beim Abfohlen), es können infektiöse Ursachen verantwortlich sein (Leptospiren, Chlamydien, Coxiellen, Pilze, BVD, Neospora, Toxoplasma, Salmonellen, Brucellen sowie Opportunisten (T. pyogenes, E. coli, Streptococcus spp)), oder aber genetische Faktoren können eine Rolle spielen. Zurzeit gibt es keine genaueren Angaben zu Prävalenzen zu Aborten und Totgeburten und den damit verbundenen Ursachen in der Schweiz resp. in Europa. Eine Studie aus Neuseeland bei Alpakas ergab einen Verlust von Föten nach 100 Tagen Trächtigkeitsdauer an - dieser lag bei 5% (Ridland M. et al. 1993). In einer anderen Studie wurden 10-17% beobachtet (Knoght TW. et al. 1995). In der Schweiz gibt es laut BFS 2016 6708 Neuweltkameliden in der Schweiz. Leider wurde nicht erfasst, wie viele davon weibliche Tiere sind. Rein hypothetisch und rechnerisch gehen wir von einer Hälfte weiblicher Tiere aus und nehmen 5% Verlust für die Berechnung an, so könnte man mit einer Maximalrate von 167 Aborten pro Jahr rechnen. Informationen zu Prävalenzen sowie (häufige) Ursachen für Aborte und perinatale Sterblichkeit sind weitgehend unbekannt, da wenig Informationen zu diesem Thema bei Neuweltkameliden publiziert worden sind. Diese Studie soll sich am Goldstandard der Studien von John Mee (Publikationen 1991-2015) orientieren und hat zum Ziel, über die nächsten 2 Jahre Problembetriebe in der Schweiz zu erfassen und genauer zu untersuchen. Die Studie wird deskriptiv als Basisstudie durchgeführt.

44.2 Konkrete Erkenntnis, die angestrebt wird

Untersuchung und Beschreibung von Aborten und Totgeburten auf Problembetrieben. Dabei besteht auch die Möglichkeit der Detektion auszurottender, zu bekämpfender und zu überwachender Tierseuchen. Mögliche genetische Letalfaktoren zu entdecken. Grundlage zur Verbesserung des Managements (Tierschutzrelevanz).

5 ANGABEN ZUR METHODE (Beschreibungen und Anmerkung zu den Ziff. 51-58)

51.1 Überblick über das Projekt (Versuchsanordnung, Übersicht über die Methode, ggf. Name des Tiermodells, Ablauf des Versuchsvorhabens, ggf. Ablaufdiagramm, Biometrische Planung) (Details zur Methode unter Ziffer 54)

Information von Nutztierärzten/-innen sowie Tierhalter/-innen von Neuweltkameliden, die uns in der Folge solche Fälle/Betriebe melden. Betriebsbesuch mit Fragebogen für Tierhalter/-innen. Untersuch von Aborten, Plazentae sowie perinatal verstorbenen Fohlen am Institut für Tierpathologie der Vetsuisse Fakultät Bern (Probenanalyse am Institut für Veterinärbakteriologie, Institut für Parasitologie). Es wird von jedem Fohlen ein Stück Ohr für das Institut für Genetik eingefroren und ein PCR für die BVD-Analyse durchgeführt. Blutproben des Muttertieres werden entnommen zur Untersuchung auf Spurenelemente und eingefroren zur möglichen späteren genetischen Analyse bzw. um Titer gegen bakterielle Infektionen nachweisen zu können. Zeitgleich auftretende Aborte im Betrieb werden mituntersucht.

Patrik Zanolari 11.04.2018 17:45

Ergänzung: Falls beim Tierarztbesuch keine Plazenta auffindbar ist, kann ein Vaginaltupfer entnommen werden.

51.2 Begründung für die Wahl der Methode oder des Modells unter Darstellung der Besonderheiten/Vorteile (Art. 137 Abs. 3 TSchV)

Prävalenzerhebung über die Gründe von Aborten in der Schweiz bei Neuweltkameliden; Problembetriebe sollten genauer analysiert werden; Basis für prospektive Abklärungen.

51.3 Begründung für die Wahl der Tierarten und falls zutreffend für das Verwenden von Tieren, die nicht zu Versuchszwecken gezüchtet wurden

Patientengut = Neuweltkameliden

52 Vorbereitung d. Tiere auf den Versuch (Eingangsunt. Art.135 Abs.3, Angewöhnung Art.119 Abs.1 TSchV, Markierungsart Art. 120 TSchV und Art.5 Abs.2 TVV, Konditionierung, Futter-/Wasserentzug, Vorbehandlung etc.)

Keine

53.1 Anästhesie und/oder weitere Schmerzbekämpfung (Mittel, Dosen, Applikationsweg und -häufigkeit, Zeitdauer etc.) (Art. 135 Abs. 5 TSchV)

Keine

53.2 Begründung für die Wahl der Anästhesie und/oder Analgesie sowie ggf. Begründung für den Verzicht auf belastungsmindernde Massnahmen

Die Blutentnahme mittels Vakutainer ist nicht anästhesiewürdig.

54.1 Art der Eingriffe/Manipulationen und Erheben von Parametern am Tier (Ablaufschema für das einzelne Tier/für die Tiergruppe angeben): operative Eingriffe (Ablauf), Substanzapplikation (Art und Ort, Menge und Häufigkeit), Infizierung, physikalische Einwirkungen (Bestrahlungen etc.), Verkaufskontrollen, Probenerhebung, Reaktionstest etc. Angaben mittels Standard Operation möglich (SOP)

SOP für die Untersuchung der toten Fohlen und Plazentae sind vorhanden; an den Müttern dieser Fohlen sind ausschliesslich Blutentnahmne an der Jugularvene geplant.

null 09.04.2018 09:34

Ein SOP für die Untersuchungen wird erwähnt, fehlt aber im Gesuch. Bitte ergänzen.

null 11.04.2018 17:41

Das Standard Operating Procedure (SOP) wurde hinzugefügt. Falls beim Tierarztbesuch keine Plazenta auffindbar ist, kann ein Vaginaltupfer entnommen werden.

54.2 Dauer der Versuchsserie (falls sinnvoll in geeigneter tabellarischer Darstellung): gesamte Versuchsdauer für jede einzelne Gruppe oder jedes Tier, inkl. Zeitdauer, während der das Tier Substanzen oder anderen Noxen ausgesetzt ist. Bei wiederholter Verwendung der Tiere den Abstand zwischen den Versuchen angeben.

Gemeldete Betriebe über 2 Jahre. Maximal 2 Blutproben pro Muttertier (1 Blutprobe zeitnah zu perinatal verstorbenem Fohlen; bei Indikation eine spätere Blutprobe zum Nachweis einer Serokonversion; 2-malige Blutentnahme im Abstand von 4 Wochen (je 10 ml Blut)).

Patrik Zanolari 11.04.2018 17:48

Falls beim Tierarztbesuch keine Plazenta auffindbar ist, kann ein Vaginaltupfer entnommen werden.

54.3 Anzahl Tiere pro Versuch/Versuchsserie: Anzahl Gruppen (inkl. alle Variablen, z.B. Dosen, Zeitdauer, Kontrollen und Angaben zur zeitlichen Staffelung der Versuche gemäss Art. 137 Abs. 4 Bst.c TSchV) und Anzahl Tiere pro Tierlinie, pro Gruppe, Geschlecht der Tiere

Abortereignisse bei Neuweltkameliden sind selten. Daher wird versucht, während der Versuchsdauer alle Aborte und perinatal verstorbenen Tiere untersuchen zu lassen. Maximal 100 Betriebe (mit 1 bis maximal 3 abzuklärenden Tiere pro Betrieb); maximale Anzahl Blutproben 600 aus maximal 300 Neuweltkamelidenmuttertieren.

54.4 Begründung für die vorgesehenen Tierzahlen pro Versuch/Versuchsserie inkl. Der statistischen Behandlung der Daten (Art. 137 Abs. 4 TSchV)

100 Betriebe entspricht der oberen Kapazitätsgrenze der Doktorandin. Es ist im Voraus unklar, wie viele Betriebesich melden resp. gemeldet werden.

55 Beurteilung der Versuchsmethode und des Modells bezüglich Umsetzung der 3R-Prinzipien (Art. 137 TSchV)

Analyse des Blutes der Muttertiere auf Spurenelemente und mögliche Titerbestimmungen sind sinnvoll. Blutentnahme mit Vakutainer an der Jugularvene ist unbelastend für das Muttertier.

Patrik Zanolari 11.04.2018 17:49

Die Entnahme eines Vaginaltupfers - nur wenn keine Plazenta vorhanden - ist unbelastend für das Muttertier.

56.1 Erwartete Auswirkungen auf die Gesundheit und das Wohlbefinden der Tiere (Allgemeinzustand, Aktivität, Futter- und Wasseraufnahme, Schmerzreaktionen, Dauer und Verlauf der Beeinträchtigungen, weitere Verhaltensparameter, Wachstum, erwartete Todesfälle, etc.)

Keine

56.2 Überwachung des Wohlbefindens der Tiere (Art.135 Abs.4 TSchV): durch welche Person(en) Häufigkeit, Beurteilungskriterien, Dokumentation (z.B. score sheet gemäss Art. 144 Abs. 1 TSchV) entsprechend der Versuchsphase

Hoftiere, normale Überwachung, keine weiteren Massnahmen notwendig, da Tiere nicht beeinträchtigt werden.

56.3 Kriterien/Angaben für belastungsmindernde Massnahmen und (vorzeitigen) Versuchsabbruch (Abbruchkriterien; Art. 135 Abs. 1 und 8 TSchV) und für Verzicht auf Wiederverwendung der Tiere

Keine

56.4 Verteilung der Tiere pro Schweregrad (Art. 30 Bst. J TVV)

alle Schweregrad 0

57.1 Nummer oder Name der Versuchstierhaltung-Bewilligung angeben (falls keine bewilligte Versuchstierhaltung, Angabe von: Haltung und Pflege der Tiere vor, während, zwischen und nach Einzelversuchen; Platzangebot, Käfigtyp inkl. Anzahl Tiere, Strukturierung, Auslauf, Einzel- oder Gruppenhaltung, Fütterung und Beschäftigung, Routinekontollen durch Tierpfleger/innen, etc.)

normale Hoftiere

57.2 Begründung für allfällige Abweichungen von den Haltungsbedingungen gemäss Tierschutzverordnung resp. der oben erwähnten Bewilligung für Versuchstierhaltung (Beispiele: Futterentzug, Immobilisation, Einzelhaltung für soziale Tierarten)

Keine

58 Tötungsmethode (Mittel, Dosen, Applikationsweg, etc.), Verwendung der Tiere nach Abschluss des (Einzel-) Versuchs (wiederholter Einsatz im gleichen bzw. in anderem Versuch)

Normale Hoftiere; keine Tötung vorgesehen.

6 ANGABEN ZUR BEGRÜNDUNG DES TIERVERSUCHS

61 Welche anderen Versuchsmethoden sind (z. B. aus der Literatur) bekannt, die es ermöglichen, entsprechende Information zu erhalten (In-vitro oder In-vivo Methoden angeben Art. 137 Abs. 2 und 3 TSchV)

Keine

62 Angabe, ob das Vorhaben begutachtet wurde/wird, und wenn ja, von welcher Institution/Organisation

Nein

---

63 Güterabwägung: Beurteilung des erwarteten Erkenntnisgewinns oder Ergebnisses im Vergleich zu der den Tieren zugefügten Belastung (Art. 3 und Art. 19 Abs. 4 TSchG).

Im Rahmen dieser Güterabwägung (Art. 26 TVV) sind insbesondere der angestrebte Nutzen gemäss Ziffern 44.1 und 44.2 und die Belastungen der Tiere gemäss Ziffern 56.1-56.4 zu würdigen und einander gegenüber zu stellen.

Mögliche Detektion von (a) infektiösen Ursachen (evtl. Tierseuchen), (b) Managementfehlern (Tierschutzrelevanz), (c) Fütterungsfehlern (Selenmangel), (d) genetische Defekten. Der Nutzen überwiegt mehrfach, da allenfalls auch Nutzen für den gesamten Bestand entsteht.

Angehängte Dateien

Datum der Bewilligung

11.05.2018

Bewilligung gültig bis

11.06.2021

Entscheid Das Gesuch bildet integralen Bestandteil der Bewilligung.

Bewilligt

Bewilligte Tiere

Tierart	Nummer
Various mammals	300

Gebühren

Beschreibung	Preis
Grundgebühr neues Gesuch SG 0	**270.0**
Zuschlag für koordinierte Bewilligung Tierversuche (mehrere Kantone)	**120.0**
Total	390.0

Kommission

Name der Person	Einladung versendet am	Erinnerung versendet am	Stellungnahme
Luca Bacciarini	23.04.2018	23.04.2018 14:21	

Marina Beaud	23.04.2018	23.04.2018 14:21	
Friedrich Beermann	23.04.2018	23.04.2018 14:21	
Friedrich Beermann	23.04.2018	23.04.2018 14:21	
Friedrich Beermann	23.04.2018	23.04.2018 14:21	
Edith Bertozzi	23.04.2018	23.04.2018 14:21	
Monique Blunier	23.04.2018	23.04.2018 14:21	
Corinne Bourquin	23.04.2018	23.04.2018 14:21	
Iris Brunhart	23.04.2018	23.04.2018 14:21	
Iris Brunhart	23.04.2018	23.04.2018 14:21	
Gaëlle Bussard	23.04.2018	23.04.2018 14:21	
Gaëlle Bussard	23.04.2018	23.04.2018 14:21	
Gaëlle Bussard	23.04.2018	23.04.2018 14:21	
Gaëlle Bussard	23.04.2018	23.04.2018 14:21	
Gaëlle Bussard	23.04.2018	23.04.2018 14:21	
Gaëlle Bussard	23.04.2018	23.04.2018 14:21	
Gaëlle Bussard	23.04.2018	23.04.2018 14:21	

Gaëlle Bussard	23.04.2018	23.04.2018 14:21	
Thomas Bürge	23.04.2018	23.04.2018 14:21	
Doris Bürgi Tschan	23.04.2018	23.04.2018 14:21	
José Cachim	23.04.2018	23.04.2018 14:21	
Gabriela Calzavara-Guldener	23.04.2018	23.04.2018 14:21	
Simona Casati	23.04.2018	23.04.2018 14:21	
Kai Caspari	23.04.2018	23.04.2018 14:21	
Isabelle Castro	23.04.2018	23.04.2018 14:21	
Isabelle Castro	23.04.2018	23.04.2018 14:21	
Isabelle Castro	23.04.2018	23.04.2018 14:21	
Isabelle Castro	23.04.2018	23.04.2018 14:21	
Isabelle Castro	23.04.2018	23.04.2018 14:21	
Thomas Christen	23.04.2018	23.04.2018 14:21	
Default CO	23.04.2018	23.04.2018 14:21	
Default CO	23.04.2018	23.04.2018 14:21	

Default CO	23.04.2018	23.04.2018 14:21	
Default CO	23.04.2018	23.04.2018 14:21	
Default CO	23.04.2018	23.04.2018 14:21	
Default CO	23.04.2018	23.04.2018 14:21	
Marie-Louise Degonda	23.04.2018	23.04.2018 14:21	
Gabrielle Garoflid	23.04.2018	23.04.2018 14:21	
Gabrielle Garoflid	23.04.2018	23.04.2018 14:21	
Gabrielle Garoflid	23.04.2018	23.04.2018 14:21	
Giuseppina Gelormini	23.04.2018	23.04.2018 14:21	
Giuseppina Gelormini	23.04.2018	23.04.2018 14:21	
Giuseppina Gelormini	23.04.2018	23.04.2018 14:21	
Simone Gilg	23.04.2018	23.04.2018 14:21	
Simone Gilg	23.04.2018	23.04.2018 14:21	
Simone Gilg	23.04.2018	23.04.2018 14:21	
Simone Gilg	23.04.2018	23.04.2018 14:21	
Simone Gilg	23.04.2018	23.04.2018 14:21	

Corinne Grandjean-Gueltzer	23.04.2018	23.04.2018 14:21	
Corinne Grandjean-Gueltzer	23.04.2018	23.04.2018 14:21	
Corinne Grandjean-Gueltzer	23.04.2018	23.04.2018 14:21	
Marco Gut	23.04.2018	23.04.2018 14:21	
Marco Gut	23.04.2018	23.04.2018 14:21	
Marco Gut	23.04.2018	23.04.2018 14:21	
Marco Gut	23.04.2018	23.04.2018 14:21	
Lucia Henchoz	23.04.2018	23.04.2018 14:21	
Markus Höfliger	23.04.2018	23.04.2018 14:21	
Nicola Jäggin	23.04.2018	23.04.2018 14:21	
Nicola Jäggin	23.04.2018	23.04.2018 14:21	
Marc Kirchhofer	23.04.2018	23.04.2018 14:21	
Michaela Käslin	23.04.2018	23.04.2018 14:21	
Michaela Käslin	23.04.2018	23.04.2018 14:21	

Michaela Käslin	23.04.2018	23.04.2018 14:21	
Michaela Käslin	23.04.2018	23.04.2018 14:21	
Monika Landtwing	23.04.2018	23.04.2018 14:21	
Michel Laszlo	23.04.2018	23.04.2018 14:21	
Claudia Lawnitzak	23.04.2018	23.04.2018 14:21	
Claudia Lawnitzak	23.04.2018	23.04.2018 14:21	
Claudia Lawnitzak	23.04.2018	23.04.2018 14:21	
Claudia Lawnitzak	23.04.2018	23.04.2018 14:21	
Claudia Lawnitzak	23.04.2018	23.04.2018 14:21	
Carmen Läubli	23.04.2018	23.04.2018 14:21	
Otto Maissen	23.04.2018	23.04.2018 14:21	
Helen Murer	23.04.2018	23.04.2018 14:21	
Rainer Nussbaumer	23.04.2018	23.04.2018 14:21	
Alexa Oppliger	23.04.2018	23.04.2018 14:21	
Romina Palazzo	23.04.2018	23.04.2018 14:21	
Romina Palazzo	23.04.2018	23.04.2018 14:21	

Manuela Pasqual	23.04.2018	23.04.2018 14:21	
Manuela Pasqual	23.04.2018	23.04.2018 14:21	
Giovanni Peduto	23.04.2018	23.04.2018 14:21	
Sascha Quaile	23.04.2018	23.04.2018 14:21	
Sascha Quaile	23.04.2018	23.04.2018 14:21	
Björn Rapp	23.04.2018	23.04.2018 14:21	
Astrid Rod	23.04.2018	23.04.2018 14:21	
Michel Rérat	23.04.2018	23.04.2018 14:21	
Michel Schmitt	23.04.2018	23.04.2018 14:21	
Nicole Schnyder	23.04.2018	23.04.2018 14:21	
Grégoire Seitert	23.04.2018	23.04.2018 14:21	
Petra Sidler	23.04.2018	23.04.2018 14:21	
Nicolas Späth	23.04.2018	23.04.2018 14:21	
Nicolas Späth	23.04.2018	23.04.2018 14:21	
Sibylle Stadelmann	23.04.2018	23.04.2018 14:21	
Barbara Thür	23.04.2018	23.04.2018 14:21	

Peter Uehlinger	23.04.2018	23.04.2018 14:21	
Cristina Villiger	23.04.2018	23.04.2018 14:21	
Lucile Vogt	23.04.2018	23.04.2018 14:21	
Lucile Vogt	23.04.2018	23.04.2018 14:21	
Lucile Vogt	23.04.2018	23.04.2018 14:21	
Paul Witzig	23.04.2018	23.04.2018 14:21	
Erika Wunderlin	23.04.2018	23.04.2018 14:21	
Walter Zeller	23.04.2018	23.04.2018 14:21	
Walter Zeller	23.04.2018	23.04.2018 14:21	
Karola Zellweger	23.04.2018	23.04.2018 14:21	
Anna Lena Zogg	23.04.2018	23.04.2018 14:21	
Anna Lena Zogg	23.04.2018	23.04.2018 14:21	
