# Supplementary material for: Causes of Abortions in South American Camelids in Switzerland—Cases and Questionnaire
Source: Animals (Basel). 2021 Jun 30;11(7):1956. doi: 10.3390/ani11071956 (PMC8300385; doi:10.3390/ani11071956)
Supplement: Supplementary file 1 [file animals-11-01956-s001.zip › animals-1248084-supplementary/List of owners of aborted crias.pdf]

List of owners of aborted crias (Rüfli et al.)

Lauber Beat, Irchelstr. 26a, CH-8428 Teufen

Herger Peter, Ribenen 3, CH-6469 Haldi b. Schattdorf

Bilang Barbara, Pestalozzistr. 57, CH-3603 Uetendorf

Marbacher Monika, Oberdorf 14, CH-6022 Grosswangen

Felder Priska, Stillaub, CH-6162 Finsterwald

Rüedi Andi, Bernstr. 170, CH-3042 Ortschwaben

von Berger Stephanie, Platti 333F, CH-6085 Hasliberg
