# Supplementary material for: Causes of Abortions in South American Camelids in Switzerland—Cases and Questionnaire
Source: Animals (Basel). 2021 Jun 30;11(7):1956. doi: 10.3390/ani11071956 (PMC8300385; doi:10.3390/ani11071956)
Supplement: Supplementary file 1 [file animals-11-01956-s001.zip › animals-1248084-supplementary/Questionnaire 1_Rüfli et al.pdf]

Abortion und perinatal mortality of

South American camelids

Questionnaire

Date of 1st contact: ________________________________

Date of 1st farm visit: ________________________________

Date of further visits: ________________________________

Personal data

Surname: ____

Name: ____

Address: ____

Phone: ____

E-Mail: ____

Veterinarian: ____

TVD – number : ____
(Farm identification number)

Farm data

Employees (besides farmer):

□ family members □ apprentice □ employees

Farm size:

Dams: ________________________________

Stud: ________________________________

animals < 1 year: ________________________________

other animals: ________________________________

Housing system:

□ pasture □ shelter □ stable □ paddock □ other: ______________

Feeding:

□ pasture □ hay □ concentrate □ other: _____________

Mineral supply:

□ minerals □ salt cup □ vitamins □ other: _____________

Trade:

□ self - restocking

□ purchase: □ Europe □ North America □ South America □ other: __________

Parturition management

Location:

□ pasture □ shelter □ stable □ paddock □ other: ______________

□ alone □ with herd

Time point of change of location :

□ sacrotuberous ligaments □ swollen vulva □ restlessness □ separation from herd
□ rupture of amniotic sac □ legs visible

Control of the dam:

□ continuous □ 30min □ 60min □ 90min □ 120min □ > 120min

Birth assistance:

Please describe in detail, how you assist the dam giving birth:

____
____
____
____

□ warm water □ soap □ Betadine □ gloves

Auxiliary means:

□ lubricant □ other: ______________

Initial care of the cria:

□ free respiratory passages □ remove "third" skin □ rub with straw □ disinfection of umbilicus

□ Selenium / Vitamin E injection □ nothing

Cleaning / disinfection:

□ after each birth □ monthly □ biannual □ annual □ never

□ broom □ hot water □ high – pressure cleaner

□ disinfection: __________________

Abortion / cria losses:

Amount of losses in 1 year: ____

Start of losses: ____

Time of losses: □ early gestation □ late gestation □ post partum

Diagnostic work up of causes: □ yes: ______________________________ □ no

Specific case data

Sire:

Name: ______________________________

Microchip: ______________________________

Species: □ llama □ alpaca

Dam:

Name: ______________________________

Microchip: ______________________________

Species: □ llama □ alpaca

Date of birth: ______________________________

Parity: ________________

Date of mating: ________________

BCS: ________________

Birth: □ spontaneous □ with aid □ induced: ________________

□ day □ night

Afterbirth: □ immediately □ after 30min □ after 60min □ > 60min

□ completely □ partially

weight: ______________

Vaccination : □ yes: ________________________ □ no

time: ____________________

Deworming: □ yes: ________________________ □ no

time: ____________________

Cria:

Species: □ llama □ alpaca

Sex: □ female □ male

Date of birth: ______________________________

weight: ________________ □ weighed □ estimated

Colostrum uptake: □ yes □ no □ amount: ______________

Time of death: □ abortion □ stillbirth □ within 1 day □ within several days

Malformation: □ hydrocephalus □ choanal atresia □ atersia ani

□ cleft palate □ ankylosis □ anasarca

birth: □ observed □ not observed

□ unassisted □ assisted by owner □ assisted by veterinarian

□ manual □ epidural □ caesarian section

Cria observed: □ birth □ laying □ standing □ dead

Duration of birth: □ 5 - 10min □ 10 – 20min □ 20 – 30 min □ 30 – 60min □ > 60min

□ not observed

Dystocia: □ inadequate cervical dilation □ torsio uteri
□ malpresentation /- position / - posture

□ absolute / relatively too large fetus

Presentation / position / posture

□ anterior presentation □ posterior presentation □ unknown

□ dorsal position □ ventral position

□ lateral deviation of head □ downward deviation of head

□ carpal flexion □ elbow flexion □ shoulder flexion

Umbilicus:

□ normal □ short □ hemorrhage □ disinfection: _________________

Health status dam:

Ante partum: □ healthy □ sick: ____

Post partum: □ healthy □ sick: ____

Previous abortion / stillbirths: □ yes, when: _______________________ □ no

Management changes in the last 3 months:

Husbandry: ____

Feeding: ____

Purchase: ____

Change of pasture: ____

Employees: ____
