# Supplementary material for: Causes of Abortions in South American Camelids in Switzerland—Cases and Questionnaire
Source: Animals (Basel). 2021 Jun 30;11(7):1956. doi: 10.3390/ani11071956 (PMC8300385; doi:10.3390/ani11071956)
Supplement: Supplementary file 1 [file animals-11-01956-s001.zip › animals-1248084-supplementary/Questionnaire 2_Rüfli et al.pdf]

# Questionnaire 2

**Herd management**

Who is the main caretaker of the herd?

□ Family members □ apprentice □ employees □ other

How many sires /geldings does the farm have?

□ 1 □ 2-10 □ >10

How many dams does the farm have?

□ 1-5 □ 6-10 □ 10 – 20 □ > 20

What other animals live on the farm?

□ none □ cattle □ dogs □ cats □ horses □ other

Are animals bought in? If yes, from where?

□ Switzerland □ Europe □ North America □ South America
□ New Zealand / Australia □ other

Are purchased animals being isolated at first?

□ yes □ no

**Parturition management**

Is the dam being observed at the time of birth?

□ yes □ no

Is the dam separated from the herd for the parturition?

□ yes □ no

How many abortions occurred on your farm in the last 2 years?

□ 1 □ 2 □ 2-5 □ >5 □ none

Was the abortion examined? Veterinarian / Laboratory / Necropsy

□ yes □ no

How was the abort material disposed of?

➔ *Short answer*
